# The Flexibility of Working Memory in Drawing on Episodic Long-Term Memory Representations in Serial Recall

**DOI:** 10.5334/joc.451

**Published:** 2025-07-18

**Authors:** Ana Rodriguez, Philipp Musfeld, Lea M. Bartsch

**Affiliations:** 1 University of Zurich, CH

**Keywords:** long-term memory, working memory, serial recall, encoding structure

## Abstract

Prior episodic long-term memory (LTM) can enhance working memory (WM) by improving recall of WM representations that match pre-learnt information and by freeing up capacity for new information. In this study, we investigated the flexibility of WM in doing so. Specifically, we tested whether WM can make use of pre-learnt item-item associations in a serial recall task, which typically requires the formation of item-positional bindings. We examined whether any benefits arise from accessing full episodic representations or from item activation, and assess whether the observed benefits are best explained by compression accounts during encoding (e.g., chunking, offloading) or by redintegration at test. Furthermore, we tested whether the benefits for pre-learnt and novel words depended on the position within the lists. Across three experiments, we consistently found that incorporating pre-learnt word pairs into a serial recall task facilitated immediate memory for words that matched pre-learnt representations – speaking against an item activation account. However, the benefit on new words within lists that included pre-learnt pairs depended on whether the words could be easily submitted to encoding strategies, such as chunking or offloading, which was facilitated by providing matching grouping structures during encoding. Overall, our results expand our understanding of how prior experiences can benefit WM processes, demonstrating that such benefits mainly result from the retrieval of prior episodes, rather than enhanced item activation in episodic memory.

When we are required to retain information for a brief period of time in order to accomplish current goals, we make use of our working memory (WM) system. Compared to long-term memory (LTM) – a system that can store extensive amount of information over long periods of time (Cowan, 2008; Squire, 2004) – WM is very limited in the amount of information that it can hold (Cowan, 2008; Oberauer, 2009).

Despite the functional differences between WM and LTM, both systems interact (Atkinson & Shiffrin, 1968; Cowan, 2008; Unsworth & Engle, 2007). In fact, current theories of WM (Cowan, 1999; Oberauer, 2009) propose that the WM system comprises activated representations in LTM that are actively bound to the current context, and one core function of WM would be to hold those bindings for task resolution (Oberauer, 2019). For instance, processing information in WM has been proposed to lead to the creation of new LTM representations (Atkinson & Shiffrin, n.d.; Bartsch et al., 2018; Cotton & Ricker, 2021, 2022), and LTM representations could help in using the limited capacity of WM more efficiently (Bartsch & Oberauer, 2023; Bartsch & Shepherdson, 2022; Chen & Cowan, 2005).

## Contributions of LTM to WM

Contributions from LTM to WM are manifold and can result from many different aspects of our knowledge system (Bartsch, Fukuda, et al., 2024). Among others, this can include contributions from our lexical and phonological system (Hulme et al., 1991; Jefferies et al., 2006; Kowialiewski & Majerus, 2018; Saint-Aubin & Poirier, 2000), which are fundamental components of language, enabling the perception and comprehension of verbal information and thus supporting its encoding and retrieval; semantic knowledge about specific facts (Ericsson & Kintsch, 1995) that allows us to comprehend complex contents, or episodic knowledge, resulting from our previous experiences (Tulving, 1993, 2002).

Here, we want to focus on the contribution of episodic memory to WM, as this is an area which, so far, has received limited attention.

Episodic memory refers to our memory for specific events, which include context information about when (temporal), and where (spatial) these events have occurred. Classically, episodic memory has been distinguished from semantic memory, which is supposed to be independent of specific contextual information (Tulving, 1972; Renoult et al., 2019). Two ways have been proposed in which episodic knowledge can contribute to WM.

First, episodic LTM representations have been argued to be immediately formed within WM, serving as the basis of new rapid learning (Cowan, 2019). These representations can be accessed instantly and remain active throughout an immediate test of WM, but their contributions to performance are more likely to occur when WM capacity has been exceeded (Bartsch & Oberauer, 2023; Oberauer & Awh, 2022).

Second, another way in which episodic LTM can contribute to WM performance is through prior knowledge. Episodic prior knowledge represents the memory of events that participants have experienced in the past. In an experimental context, it is typically studied and manipulated through a learning phase prior to the WM task (e.g. Chen & Cowan, 2005). Here, participants are required to learn new associations that later on are included in WM trials.

## Proposed mechanisms underlying the contributions of pre-learnt episodic LTM to WM performance

Including pre-learnt LTM information – which participants learn at the beginning of an experiment – within a test of WM has been demonstrated to benefit WM performance (Bartsch & Shepherdson, 2022; Chen & Cowan, 2005, 2009; Norris & Kalm, 2021; Thalmann et al., 2019), especially when WM load is high (Bartsch, Frischkorn, et al., 2024). Such a contribution of episodic LTM to WM has been shown to benefit immediate memory performance in two ways: First, immediate memory for information presented in a task that matches a stored episodic representation is better compared to novel information (Bartsch, Frischkorn, et al., 2024; Bartsch & Shepherdson, 2022, 2023; Chen & Cowan, 2005, 2009; Norris et al., 2020; Thalmann et al., 2019). Second, apart from better memory for the pre-learnt information itself, the presence of such pre-learnt information can free-up capacity for novel information (Bartsch & Shepherdson, 2022, 2023; Musfeld et al., 2024; Norris et al., 2020; Thalmann et al., 2019).

It is assumed that when pre-learnt LTM representations are included in trials of a WM task, benefits on novel information are due to a reduction in WM load at encoding (Norris & Kalm, 2021). Two main mechanisms have been discussed in the literature to account for this reduction in WM load: chunking and offloading. When pre-learnt information is recognized, chunking strategies could arise, allowing the information to be re-encoded or compressed into smaller units (Brady et al., 2009; Chekaf et al., 2016; Chen & Cowan, 2005; Norris & Kalm, 2021; Thalmann et al., 2019). Specifically, the multiple elements of an LTM representation could be compressed and integrated in a unified representation (Brady et al., 2009; Chekaf et al., 2016), or individuals could selectively encode just pieces of the information and retrieve the associated information directly from LTM at test (Chen & Cowan, 2005; Musfeld et al., 2024; Thalmann et al., 2019). This results in a reduced necessity for encoding the information in its original presentation in WM, hence freeing-up resources. Alternatively, the information could be offloaded to LTM (Bartsch & Shepherdson, 2022, 2023; Cowan, 2008; Huang & Awh, 2018; Ngiam et al., 2019; Schurgin et al., 2018). Here, pre-learnt and accessible information in LTM would not be encoded into WM at all, but is only retrieved at test given a recall cue, thereby freeing-up WM resources for encoding other information into WM.^1^

In contrast, in case benefits arise solely for information in WM that matches pre-learnt knowledge stored in LTM, but no freeing of capacity is observed, a third alternative explanation has been proposed: redintegration. Redintegration is a process occurring at retrieval (rather than during encoding), where available LTM representations help to reconstruct degraded information held in WM (Hulme et al., 1991, 1997; Jones & Farrell, 2018). According to this account, the presence of LTM information does not alter WM representations during encoding (like it is the case for chunking or offloading), but it helps to rebuild a degraded WM representation during recall. This is more likely to be successful, when the LTM representation is strong and easily accessible (Schweickert, 1993; Thorn et al., 2005). Thus, redintegration entails that LTM information is encoded and maintained as originally presented (Norris et al., 2020), and does not predict a freeing-up of capacity in WM. Rather, the immediate memory benefit is limited to the pre-learnt LTM over novel information because it is more likely to be reconstructed during testing.

## The role of structure and representations – how flexible can WM draw on LTM?

In previous studies on the topic using verbal representations, both, the information that participants learnt prior to the WM task, as well as the stimuli presented in the WM task itself, had the same encoding structure (i.e. simultaneous presented item-item associations in form of word pairs; Bartsch & Shepherdson, 2022, 2023; Chen & Cowan, 2005, 2009).

The use of the same encoding structure might have facilitated the recognition of LTM information, making it easier to engage in strategies that ultimately reduced the amount of resources required for encoding LTM word pairs (Bartsch & Shepherdson, 2022, 2023; Chen & Cowan, 2005, 2009). This, in turn, could have caused the observed freeing of capacity (the benefits on novel information), when the lists were a mix of pre-learnt and new representations (Bartsch & Shepherdson, 2022, 2023).

While the aforementioned research has provided insight into when and how WM draws on LTM when WM and LTM representations are matched in structure, a question remains: How does WM flexibly draw on prior knowledge stored in episodic LTM when the stored representations do not match the structure of representations required for a WM task? These circumstances can be created by breaking up the representational structure of pre-learnt information when used in a WM task. Specifically, when episodic LTM representations of word pairs comprise an item-item structure (e.g., Chen & Cowan, 2005, 2009), and the information presented in serial recall tasks leads to the creation of representations of item-positional bindings (Burgess & Hitch, 1999; Henson, 1999). So far only two studies have investigated the benefit of pre-learnt item-item associations in such a case – on immediate serial recall.

On the one hand, Norris et al., (2020) used a serial recall task with mixed lists that included words that matched pre-learnt associations in LTM and newly encountered singletons (i.e., novel words, not paired with another word). They found that superiority in performance of lists including pre-learnt word pairs was driven predominantly by better memory for the LTM information itself, consistent with a redintegration account. At the same time, there was no clear advantage for singletons within the same list, except for the condition in which the lists included three word pairs. This advantage was attributed to the constrained serial positions in which singletons could appear when more word pairs were included within the list.

On the other hand, Thalmann et al. (2019, Experiment 1) presented participants with WM trials of two independent word lists (of 2 or 4 items), each presented sequentially, followed by serial recall of each list in random order. When one list consisted of pre-learnt associations of either two or four elements, there was evidence for a reduction in WM load – namely, the other list comprising of novel stimuli was remembered better compared to a condition where both lists where novel. Hence, freeing-up of WM capacity was observed.

Although converging on evidence that at least the pre-learnt LTM information is remembered better than new words in an immediate serial recall task, these previous studies leave open the question of what information WM is drawing on when utilizing pre-learnt item-item episodic LTM representations, and under which circumstances this allows to free-up capacity for other items in WM.

Referring back to conceptualizations of the interaction of WM and LTM (such as Cowan, 1999; Oberauer, 2009), and in the context of serial recall, where pre-learnt item-item episodes are disrupted by the sequential presentation of the single elements, WM could draw on LTM in two ways: 1) via the activation of item-level information in LTM, and 2) through the binding of the activated representation to the correct serial position within the list. This means, that the WM system could draw on two types of information: (1) the item activation of *each* word in LTM individually or (2) the entire episode entailing the associations between the items once the words have been presented.

Depending on which of these WM can actually draw on, the processing of the information will have different implications that ultimately underly the benefit of pre-learnt episodic LTM on WM performance. If WM relies primarily on the activation of the individual LTM items, regardless of whether they were part of pre-learnt item-item representations, these items will have a higher level of activation compared to novel items, making them easier to redintegrate them at recall. However, if WM primarily draws on the entire episode (the elements including their episodic binding) the representation could be submitted to chunking or offloading, which in consequence will free-up WM capacity for processing novel information.

## Locus of the benefits on novel information within WM

Lastly, past research has shown that benefits of contribution of LTM to WM are dependent on the position of the LTM information within the list (Mizrak & Oberauer, 2022, Thalmann et al., 2019, but see Bartsch & Shepherdson, 2023): In case WM can draw on the full episodic representation and the information can be chunked or offloaded, it has been observed that a reduction in WM load occurs for new information being presented following – not preceding – LTM information. These so-called *proactive benefits* particularly occur in case the LTM information is presented at the very beginning of the list. It has been proposed that this effect occurs because information already stored in WM experiences interference from the individually encoded LTM elements before the latter can be chunked or offloaded from WM.

In summary, it is unclear whether WM relies on the entire pre-learnt episode for small item-item representations, such as word pairs in intermixed lists for serial recall, after each word is presented sequentially, or if it draws from the activation of each associated word independently.

Additionally, if WM can flexibly draw on the pre-learnt episodes for serial recall, the information can be subjected to encoding strategies such as chunking or offloading. The use of these strategies can effectively free-up WM capacity, a benefit not observed with redintegration. However, it is unclear in this context, if a reduction in WM load is influenced by the exact position of the episode within the list, as offloading and recoding are likely more effective when LTM information is presented at the beginning of the list. To address this, it is necessary to control and counterbalance the serial positions of sequentially presented LTM words within the lists.

## The present study

The present study aimed to investigate the extent to which WM can flexibly and effectively utilize prior episodic item-item associations to benefit WM performance, particularly when the WM task requires to remember item-positional bindings.

Our first goal was to investigate whether WM benefits from item activation in LTM, which would show a general benefit for words studied during a learning phase independent of the binding between them; or whether participants benefit from pre-learnt associations by drawing on a representation containing the episodic binding – even when the WM task requires the formation of new item-positional bindings.

Our second goal pertains to examining whether the inclusion of LTM representations that consist of pre-learnt item-item association can free-up WM capacity, depending on the position in which LTM word pairs were introduced within the list.

Across three experiments, participants underwent a LTM learning phase in which they were asked to memorize word pairs (item-item associations) before performing an immediate serial recall task. The serial recall task included lists of words presented individually in sequence. Critically, these lists included words presented in succession matching specific word pairs from the LTM learning phase (*word pairs* condition; item-item associations available), or two words in succession from different word pairs (*singletons* condition; high familiarity, but no item-item associations available).

If WM can draw on LTM representations encompassing a bound episode (i.e. a word pair), then immediate memory performance in which two words from a pre-learnt word pair are presented in succession should lead to higher overall recall accuracy compared to new words of the same trials (*within* the condition), and compared to new words presented in the same serial positions of trials in which only new words are presented (*between* conditions). If, however, WM flexibly benefits via the activation of single words based on their prior familiarity, then both conditions containing pre-learnt words should show improved performance of LTM words compared to new words *within* the same condition and compared to the condition with just new words at matching serial positions.

We expect a proactive benefit for new words, in case participants are indeed able to draw on the episodic representation during the WM task. This means that the benefit of LTM to WM should be predominant for conditions in which the LTM -word pairs are presented at the very beginning of the list. At this point, encoding of the individual LTM elements does not interfere with any previously encoded information, and chunking or offloading the information should reduce WM load and free-up capacity for upcoming new information. Alternatively, if the benefit of including LTM information (word pairs or singletons) merely manifests as improved performance for that information alone, we would not expect to observe a corresponding benefit for novel information, and the benefit would be best explained by a redintegration account occurring at recall.

## Experiment 1

The goal of this Experiment was to investigate how flexible WM can rely on prior LTM information to enhance serial recall performance, either through item activation or by retrieving the entire episodic representation. Furthermore, by manipulating the positions in which LTM words were presented in succession within lists, we aimed to test if including LTM information consistent with pre-learnt episodes can free-up WM capacity selectively pro- or retroactively; or whether including LTM representations only improves immediate performance of those representations themselves, irrespective of the position in which they were presented within the list.

### Method

#### Open Practices Statement

This study was not preregistered. Materials, data, and analysis scripts for the experiments are available on the Open Science Framework at: https://osf.io/r8tyc/.

#### Participants

We recruited 93 participants online via Prolific (M_age_ = 28.46 years), who indicated English as their first language, were from English speaking countries and had an approval rate between 90-100. However, only 62 participants met the inclusion criteria for analysis, which required that they successfully learnt at least 70% of the word pairs presented during the initial phase of the experiment. Participants of this and the following Experiments gave informed consent prior to the study. We chose the initial sample size of n = 60 for this experiment because it was sufficient to detect the effects of interest in a similarly complex previous within- subject design. Due to the use of Bayesian Statistics, the sample size could have been increased in case the evidence was ambiguous (Rouder, 2014). We considered a Bayes Factor (BF) > 3 as sufficiently informative to distinguish between the main hypothesis of interest (Kass & Raftery, 1995). The experiments were carried out in agreement with the rules of the Ethics Committee of the Faculty of Arts and Sciences of the University of Zurich and did not require special approval.

#### Materials and procedure

**Figure 1:**
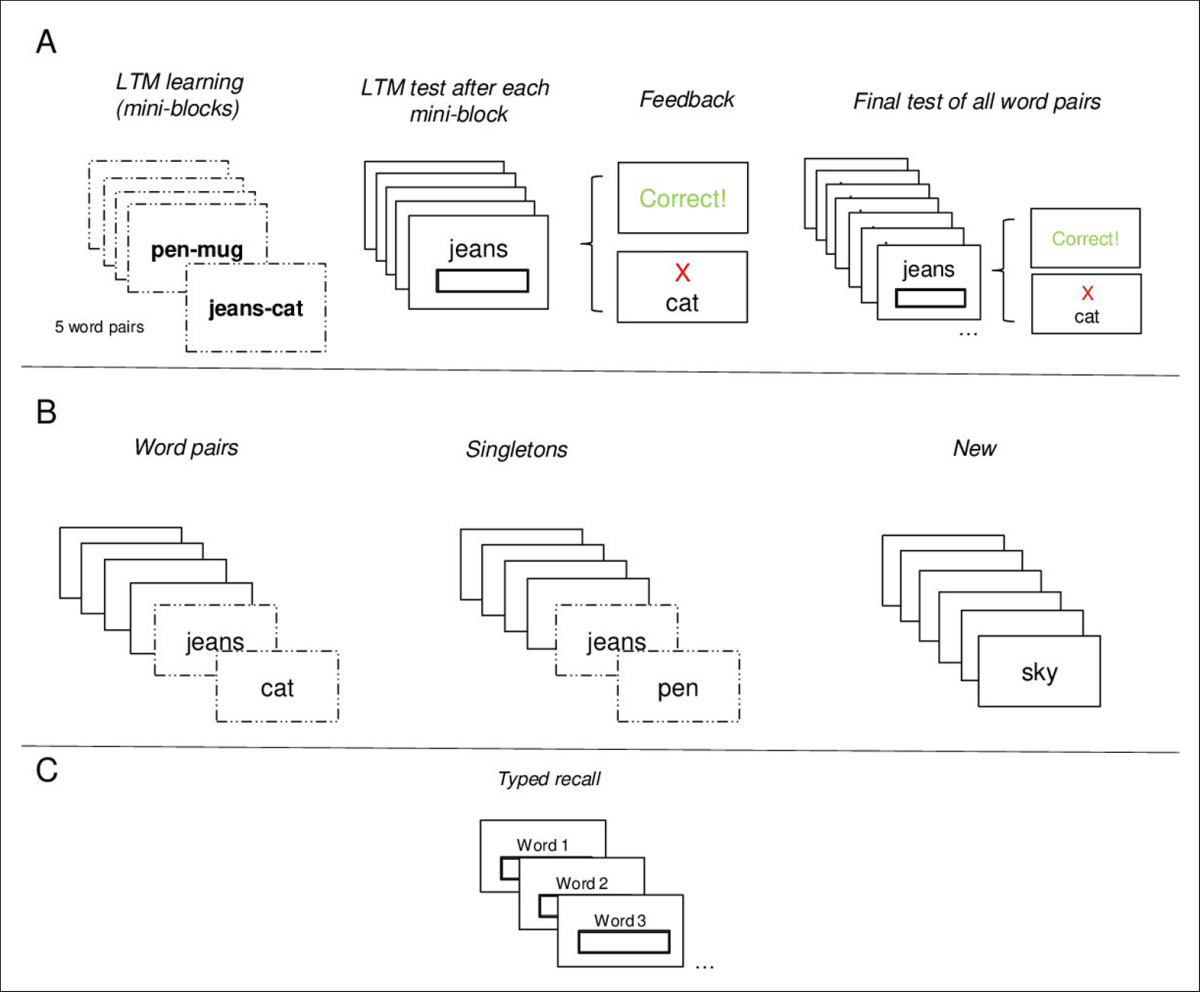
Events representing the Procedure of Experiment 1. Panel A: LTM learning Phase. Panel B: WM encoding Phase; three different Conditions from Left to Right: **(a)** LTM Word Pair **(b)** LTM Singletons from different Word Pairs **(c)** New Words. Panel C: WM testing Phase: Typed Recall *Note*. Doted frames represent the LTM words.

All experiments were programmed in jsPsych (de Leeuw et al., 2023) using the jspsych-psychophysics plugin (Kuroki, 2021). Figure 1 provides an overview of the general procedure of Experiment 1. It consisted of two main phases: an LTM learning phase and a WM task phase. Stimuli consisted of 320 English nouns chosen randomly for each participant from a set of 1192 nouns. Words were concrete, had a minimum and maximum length of 3 and 7 letters, and had an average frequency of 48.57 (*SD* = 94.11) according to the recommended Hyperspace Analogue to Language -HAL -criterion (Balota et al., 2007). Out of the 320 words, 40 were used to form 20 LTM word pairs.

As depicted in Figure 1A, the experiment started with the LTM learning phase, which consisted of the presentation of 20 word pairs divided into 4 mini-blocks. Each mini-block sequentially displayed 5 word pairs at the centre of the screen for 3500 ms (e.g., jeans-cat) with an inter-stimulus interval (ISI) of 500 ms. This was followed by a test of each word pair in random order, in which the first word of each pair was presented as a probe, and participants were required to type in the associated word. After participants pressed the “enter” key, feedback was provided. If the response was correct, a message displaying “Correct!” in green appeared immediately. If the response was incorrect, an “X” with the correct word shown in red appeared instead. Feedback lasted 2000 ms. This process was repeated a second time, with a new random order of the word pairs across the mini-blocks. Finally, participants completed a final test for all the 20 word pairs in random order following the same test procedure described earlier. These testing phases interspersed with the study were intended to boost memory through the testing effect (see Sutterer & Awh, 2016 for a similar approach), as testing has shown to improve the retention of information on the long-term (Roediger III & Karpicke, 2006).

The WM task phase consisted of the immediate serial recall of 6 words presented sequentially, each for 1000 ms with an ISI of 500 ms. This phase was divided into 3 blocks, each consisting of 20 trials. Each block represented one of three conditions: The *word pairs*, the *singletons* and the *new* condition. Blocks and thereby the order of conditions were counterbalanced across participants. In the *word pairs* condition an LTM word pair was presented in succession within the list, retaining the order in which it was originally learnt (e.g. jeans – cat). The position of the word pair was manipulated across trials, so that across the entire experiment, it appeared twice at each possible position within the list (e.g., at position “1,2”, the first word of the pair was presented at serial position 1, and the second associated word at serial position 2). In the *singletons* condition two single words of two different episodes (i.e., LTM word pairs) were presented in succession within the list (e.g. jeans – rope). As in the *word pairs* condition, the position in which the words appeared within the list was counterbalanced across trials. In the *new* condition, only new words were presented. This served as our baseline condition to measure any beneficial effects of episodic LTM to the WM task (see Figure 1B).

After the presentation of a word list, immediate memory for serial order was tested. For half of the trials, immediate memory was tested via a typed recall test, while for the other half, a 12-alternative forced choice (12-AFC) recognition test was employed. The results of the 12-AFC recognition can be found in the supplementary materials. In brief, results are consistent with the findings of the typed recall test that are described below. The tests were inter-mixed within a block. For the typed recall test, participants were required to type each word in a box at the centre of the screen, in the same order as they were presented. Each box provided a cue indicating the word to type (e.g., “Word 1”), and after typing the word and pressing “enter”, a new empty box with the proper following cue appeared (e.g., “Word 2”). Recall requires retrieval of specific and contextual details (Yonelinas, 2002), which makes the test specifically sensitive to detecting benefits of information presented as an intact episode (i.e., in the *word pairs* condition).

### Data Analysis

All analyses were conducted in a Bayesian framework using R, version 4.3.2 (R Core Team, 2023 with the following main R packages: *tidyverse* (Wickham et al., 2019), *brms* (Bürkner, 2017), *bayestestR* (Makowski et al., 2019). Data was analysed on a trial by trial basis, using the number of correct responses out of the total number of responses within a trial as the dependant variable. Correct responses were defined as accurately recalling a word in the position it was originally presented in –*strict recall*.

To account for typos in responses, we used the *stringdist* package (Van der Loo, 2014) in R with the Damerau-Levenshtein distance method. This allowed us to measure the similarity between a participant’s response and the correct answer by computing string distances. Responses with a distance of less than 2 were classified as correct.

To quantify the relative evidence in favour or against our hypotheses of interest, we computed BFs for nested models. If one model includes a parameter reflecting an effect of interest (M1), and a competing model omits this parameter, the BF can be used to quantify the relative evidence in favour or against this effect given the observed data. For instance, a BF_10_ = 10 for a model including an effect of interest against a model omitting this effect of interest, would mean that the data are 10 times more likely under the model including the effect, compared to a model omitting the effect. Conversely, we can calculate the evidence against the presence of an effect, by computing the reciprocal of BF_10_. Hereby, BF_01_ =1/ BF_10_ would indicate that the data are ten times more likely under the model omitting the effect over the model including the effect. Here, we used the Savage-Dickey density ratio method as an approximation of a nested model comparison for estimating the BF (Wagenmakers et al., 2010). The Savage-Dickey density ratio is calculated as the ratio between the prior and the posterior density of a parameter of interest (i.e., the effect of interest) at a theoretically interesting value. Here, this ratio is calculated for each parameter of interest at the value of 0, as this allows to quantify the evidence that the estimated parameter is different from 0, thereby indicating the presence or absence of an effect.

We implemented Bayesian hierarchical logistic regression models in *brms* (Bürkner, 2017) to estimate the binary accuracy of strict recall (correct/incorrect). We assumed a Binomial distribution predicted by the model through a logit link function. For all the analyses across the experiments, we included the maximal random-effects structure including random intercepts, as well as random participant effects for all the fixed-effects and their interactions in the model. Cauchy priors with a location parameter of 0 were assigned to all effect parameters. To assess the robustness of our results, we varied the scale of the Cauchy prior (.25, .5, .75, & 1) and re-estimated the BFs five times to guarantee results’ stability. From this, we report the median of the BFs along with the minimum and maximum values.

To test whether there were credible differences between the LTM and new words *within* both LTM conditions (*word pairs* and *singletons*), we fit a model where condition [*word pairs, singletons and new*] and word type [*LTM, new*] were included as fixed-effects and then computed the conditional effects for the pairwise comparisons of interest (LTM vs. new words within both the *word pairs* and *singletons* conditions). This allowed us to test whether there was credible evidence in favor of an immediate performance benefit for LTM words over new words within trials and whether this was due to a *local* advantage for episodic representations (benefits only for LTM words that matched pre-learnt *word pairs*) or to item activation of the words independent of their pre-learnt binding (benefits for both LTM words in the *word pairs* and *singletons* conditions).

To test whether there were differences for new words *between* conditions at each serial position of interest (i.e. proactive and/or retroactive benefits of LTM on WM), we fit three independent models using serial positions [*1,2*; *2,3*; *3,4*; *4,5*; *5,6*]; and condition [*word pairs, singletons and new*] as fixed-effects. We then computed the conditional effects for the pairwise comparisons of interest that will be described next.

The first model aimed to compare performance for LTM words of the *word pairs* and *singletons* conditions to performance of words of the *new* condition at the same serial positions in which the LTM words were introduced. For instance, immediate memory performance for remembering the LTM words presented at serial positions 1 and 2 to words presented at matching serial positions 1 and 2 in the *new* condition. This allowed us to test whether an immediate performance benefit for LTM words over new words between conditions was due to a *global* advantage for episodic representations (an advantage for LTM words that matched pre-learnt *word pairs* over words from the *new* condition at the same serial positions) or to item activation of the words independent of their pre-learnt binding (an advantage for both LTM words in the *word pairs* and *singletons* conditions over words from the *new* condition at the same serial positions).

The second and third model aimed to test whether there was a reduction in WM load by including LTM words in WM trials, depending on where the LTM words were introduced within the list. Specifically, the second model tested whether there was a proactive benefit for words introduced *after* the presentation of LTM words. For example, if LTM words were presented at positions 1 and 2, we analyzed performance differences of words at positions 3, 4, 5, and 6 across *all* conditions, instead of comparing performance differences to the aggregated performance of all words of the *new* condition. This allowed for an unbiased comparison focusing only on the serial positions of interest between conditions. The third model aimed to test whether there was a retroactive benefit for words introduced *before* the presentation of LTM words. Similarly to the second model, we analyzed performance differences across conditions at the serial positions of interest. Hence, if LTM words were presented at serial positions 5 and 6, we analyzed performance differences of words at positions 1, 2, 3 and 4 across all conditions.

### Results

**Figure 2:**
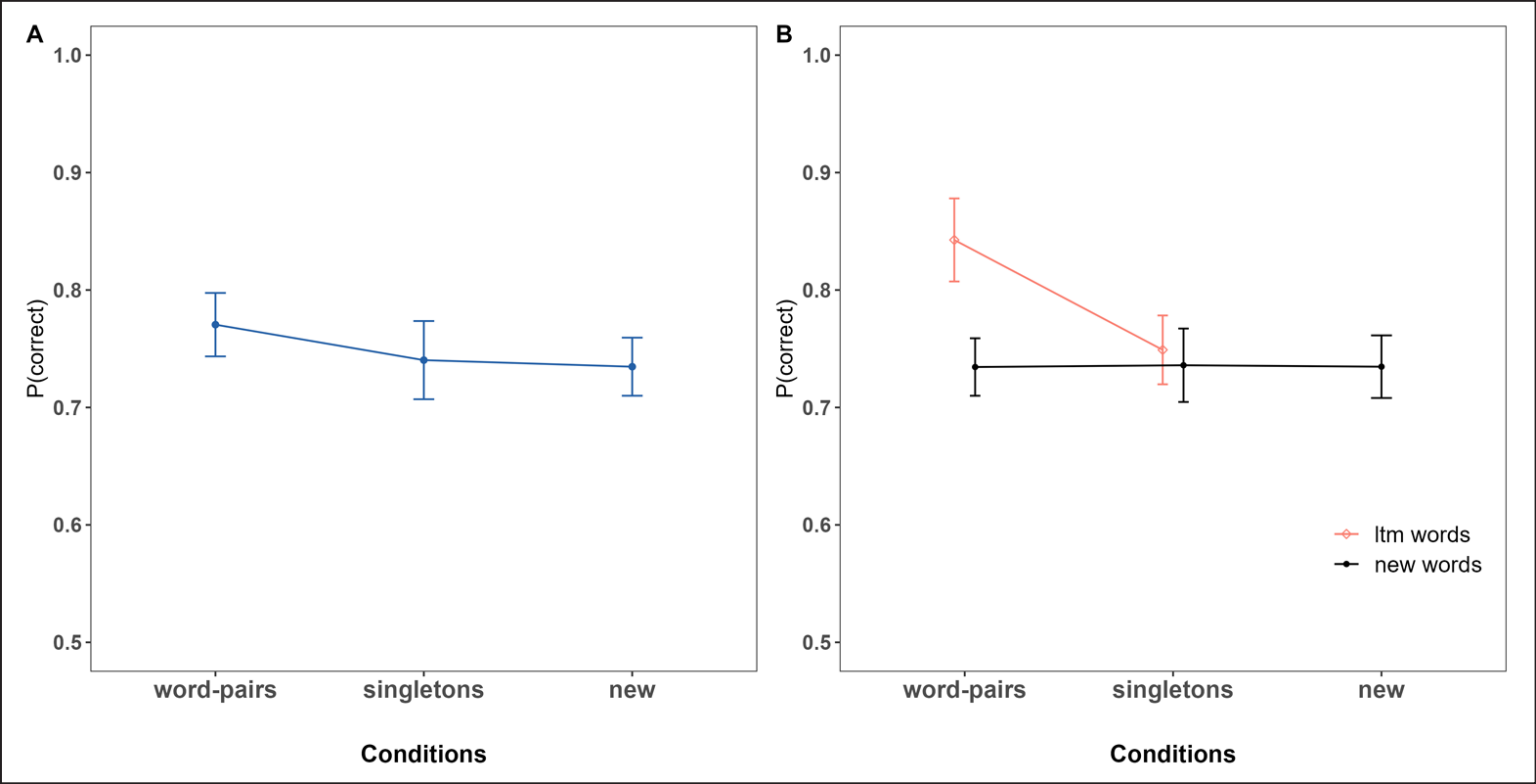
Proportion of immediate Recall Performance as a Function of Condition. **Panel A:** Overall Performance. **Panel B:** Performance for LTM and new Words for LTM conditions.

Figure 2 shows the proportion of correct responses as a function of (A) condition and (B) for LTM words and new words separately, within the *word pairs* and *singletons* conditions. As can be seen in Figure 2B, and supported by the analysis, we observed that immediate memory for LTM versus new information *within* trials was superior for LTM words that match a pre-learnt word pair in the *word pairs* condition (BF_10_ = 1.18 × 10^5^ [1.92 × 10^4^ – 1.15 × 10^11^]). However, in the *singletons* condition, when LTM information consisted of two words from different episodes, immediate memory for LTM information was not better compared to new words of the same trials (BF_10_ = 0.12 [0.07–0.28]).

This means that LTM information included in WM trials benefited immediate memory performance only when the two successive words matched an entire episode stored in LTM. In other words, LTM does not benefit serial recall performance through item activation of each singleton, but rather via the stored binding of the LTM episode.

**Figure 3:**
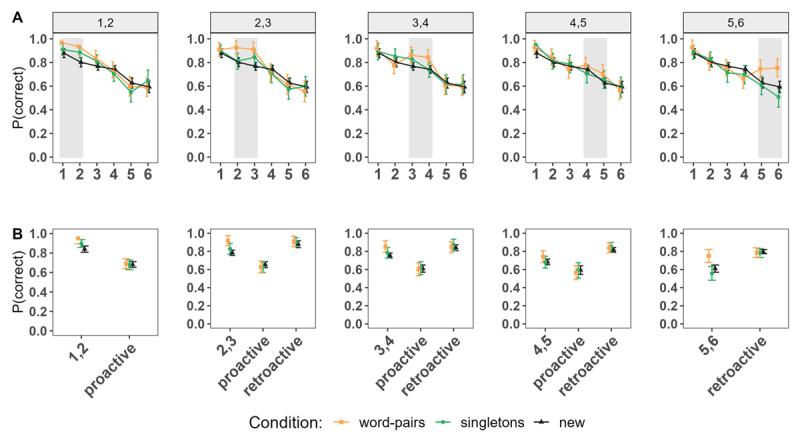
Experiment 1. **Panel A:** Serial Position Curves depending on the Serial Positions in which the LTM words were introduced within the List. **Panel B:** Performance of Words at the Serial positions of interest (LTM Words in the word pairs and singletons Conditions, Words at matching Positions of the new Condition); proactive and retroactive Performance. *Note*. The numbers (e.g., 1,2) denote the serial positions at which the LTM words were introduced within the list in both LTM conditions, and correspond to the same positions of words in the new condition. Proactive and retroactive effects indicate the aggregated performance of words presented in serial positions following or preceding the introduction of LTM words, respectively. These specific serial positions are the same for words in the new condition to ensure an unbiased comparison.

Next, we examined performance *between* conditions for LTM, as well as for new information, in order to determine global effects of LTM information, as proactive and retroactive effects depending on the position in which LTM information was presented within the list. Figure 3A shows the serial positions curves for all conditions based on the position of the LTM words within the lists. Figure 3B depicts the comparisons of interest: LTM information of *words pairs* and *singletons* conditions compared to words matching the same serial positions of the *new* condition, and performance of new words following (proactive effect) or preceding (retroactive effect) LTM information (or the matching serial positions of the *new* condition) within the list.

Supported by the statistical results presented in Table 1, we found a consistent pattern of superior performance for *LTM words* (in the *word pairs* condition) at any position within the list compared to words of the *new* condition at the same serial positions, except at positions 4 and 5. When comparing the *word pairs* to the *singletons* condition, there was anecdotal evidence of superior performance for LTM words in the *word pairs* condition when introduced at the beginning of the list (at positions 1 and 2, 2 and 3) and credible differences at the very end of the list.

**Table 1:** Experiment 1. Bayes factors (BF_10_) of the pairwise comparisons between conditions for each serial position in which the LTM words were introduced in the lists.


	*WORD PAIRS VS SINGLETONS*	*WORDS PAIRS VS NEW*	*SINGLETONS VS NEW*

*Serial positions 1,2*	1.44 [0.89–3.39]	**2041.03** [730.03–7191.33]	**4.51** [3.18–5.09]

*Proactive*	0.08 [0.05–0.18]	0.06 [0.03–0.15]	0.09 [0.06–0.22]

*Retroactive*	–	–	–

*Serial positions 2,3*	2.51 [1.66–6.01]	**776.12** [394.52–1860.73]	0.53 [0.34–0.76]

*Proactive*	0.06 [0.04–0.15]	0.07 [0.04–0.18]	0.09 [0.05–0.21]

*Retroactive*	0.17 [0.11–0.32]	0.07 [0.14–0.36]	0.35 [0.23–0.58]

*Serial positions 3,4*	0.82 [0.45–2.35]	**24.06** [17.96–48.80]	.24 [0.13–0.47]

*Proactive*	0.08 [0.05–0.18]	0.06 [0.03–0.14]	0.09 [0.05–0.20]

*Retroactive*	0.16 [0.10–0.28]	0.12 [0.07–0.21]	0.54 [0.41–0.66]

*Serial positions 4,5*	0.21 [0.10–0.83]	0.34 [0.17–1.40]	0.08 [0.04–0.19]

*Proactive*	0.11 [0.06–0.22]	0.09 [0.06–0.19]	0.11 0.07–0.24]

*Retroactive*	0.11 [0.07–0.22]	0.10 [0.06–0.23]	0.43 [0.27–0.73]

*Serial positions 5,6*	**27.25** [15.27–41.91]	**18.65** [9.01– 37]	0.13 [0.07–0.27]

*Proactive*	–	–	–

*Retroactive*	0.08 [0.05–0.18]	0.06 [0.03–0.15]	0.09 [0.05–0.23]


*Note*. Serial positions represent the positions in which the LTM words were introduced in the *word pairs* and *singletons* conditions, which are compared to the words presented at the same serial positions of the *new* condition. Credible BFs are printed in bold.

The comparison of two words of different episodes (*singletons* condition) to the *new* condition only yielded an advantage for the former, when the words were presented at the very beginning of the list (positions 1 and 2).

Regardless of where the LTM words were introduced within the list, whether matching an episode or as singletons, no proactive or retroactive benefit was found for the new words included in the list, meaning that immediate memory performance for new words across conditions was similar irrespective of whether the LTM words were presented before or after them. This suggests that including LTM words at any position of the lists did not free-up WM capacity –meaning it did not result in any advantage or disadvantage for the rest of the words included in the lists.

### Discussion

The goal of Experiment 1 was to investigate the flexibility of WM in utilizing LTM representations during serial recall. We aimed to determine whether WM can draw on entire LTM episodes or benefit from the independent activation of each element in the LTM representation when encountered individually. Additionally, we examined whether serial position influences any advantage of LTM words, either through redintegration or reducing WM load.

Our results revealed two main findings: First, we observed superior immediate memory performance for LTM information over new information *within* and *between* conditions, consistently only for words belonging to LTM episodes (i.e., pre-learnt word-pairs). Second, incorporating LTM words did not free-up WM capacity, as performance did not improve for novel words of the same lists, regardless of the serial positions of the LTM words.

The advantage of LTM words over words of the *new* condition was evident when two words from the same LTM episode were sequentially presented compared to introducing two words from different episodes. This speaks against an item activation account and is against our prediction that activating items whether from the same or different episodes would lead to higher performance via redintegration. Instead, it suggests that episodic LTM can benefit WM, when participants can retrieve *intact* episodic information for serial recall.^2^ Although participants seemed to benefit from matching episodes at any position, there was no benefit for subsequent new information, even when presented at the beginning of the list. This contrasts with previous research showing that semantic chunks introduced at the list‘s onset can free WM capacity (Portrat et al., 2016; Thalmann et al., 2019), but is consistent with previous findings that WM capacity is not freed-up during encoding when two-item chunks are included (Norris et al., 2020).

Overall, the evidence from Experiment 1 is inconsistent with both, the idea that pre-learnt information is chunked or offloaded during encoding, as we did not observe any benefit on new information within mixed lists including LTM information; and the idea that it benefits via redintegration at recall, as our results show that WM is only benefitting from the retrieval of entire episodes but not from item activation.

One possibility to resolve these inconsistencies is to assume that episodic LTM contributions arise from redintegration at recall only for words stored in LTM as full episodes rather than as individual words, thereby facilitating the redintegration effect for intact pairs but not singletons. A trial, which contains an intact pre-learnt word pair could make it more likely that at least some information about this pair is still available at test, thereby increasing the chance for its successful redintegration. Our findings would be consistent with this account, where the advantage is specific to pre-learnt episodes, but there is a lack of a benefit on novel words within the lists (Norris et al., 2020).

Another possibility could be that the current task made it more difficult to properly engage in freeing-up capacity strategies (i.e. chunking or offloading). The current paradigm requires that participants remember each word bound to their positional context. This may limit the usefulness of LTM item-item associations, as recalling each word’s position remains necessary to successfully completing the task. Thus, WM cannot exclusively rely on differently structured LTM traces, limiting the utility of freeing-up capacity strategies at encoding.

A critical difference between this and previous studies in which episodic knowledge improved WM performance is the level of facilitation in recognizing LTM information. Previous studies used tasks that inherently facilitated the recognition of pre-learnt episodes during encoding by utilizing the same item-item structure (Bartsch & Shepherdson, 2022, 2023; Chen & Cowan, 2005, 2009) or clearly separating pre-learnt information from new information (Thalmann et al., 2019). In our study, words that matched prior knowledge were less salient. By presenting them centrally like new words, we potentially reduced the effectiveness of chunking or offloading strategies that are ought to occur at encoding. Therefore, an alternative explanation for the lack of freeing-up WM capacity benefits in our results is that the different structures of LTM and WM representations made it harder to immediately recognize matching pre-learnt episodes. As a result, participants may have had to initially encode both words independently, limiting their ability to engage in encoding strategies that could otherwise reduce WM load.

Indeed, recent evidence highlights the role of awareness at encoding in WM for retrieving episodic memory. Specifically, a recent study on Hebb repetition learning (Musfeld et al., 2023), demonstrated that participants only benefited from repetitions once they had recognized a repeating episode during re-encoding. Thus, recognizing previously encountered information during WM encoding is critical for learning and retrieving LTM information.

In summary, the different representational structures used in WM and LTM might have prevented participants from recognizing and subsequently chunking or offloading redundant information during encoding. Instead, they likely encoded the information as item-context bindings, preventing the freeing of WM capacity and resulting in no advantage for new information. We test this possibility in Experiment 2.

## Experiment 2

In Experiment 2 we tested the hypothesis of whether increasing the saliency of LTM information in the WM task would increase recognizability and the beneficial contributions to WM performance. This aimed to not only improving immediate serial recall performance for the LTM words compared to new words (as in Experiment 1) but also free-up WM capacity at encoding. To address this, we highlighted the respective LTM words at encoding in red and informed that the colored words *could* match words that they had previously learnt. To control for effects of saliency, we presented two successive new words in the *new* condition in red as well, counterbalancing the serial positions across the trials.

### Method

#### Participants

An independent sample of 56 participants with an approval rate between 90-100, who indicated English as their first language and living in English speaking countries, were recruited online via Prolific. However, only 37 participants (M_age_ = 27.8 years) met the inclusion criteria for analysis, which required that they successfully learnt at least 70% of the word pairs presented during the initial phase of the experiment.

#### Materials, Procedure and Data Analysis

Materials and Procedure were equivalent to those used in Experiment 1, with the following few exceptions: First, we increased the set size of the word lists for the WM task to 7, to create more room for improvement in performance. The increase in set size meant that LTM words could appear at two extra positions within the list (serial positions 6 and 7). To ensure that the LTM words appeared an equal number of times in all the possible positions within the WM lists, participants were required to learn a total of 24 word pairs during the LTM phase. This approach helped maintain a balanced distribution of LTM word repetitions across different positions in the WM lists, similar to Experiment 1. Finally, and most importantly, the LTM words were now highlighted in red in both the *word pairs* and *singletons* conditions. We informed participants at the beginning of the experiment that the highlighted words *could* indicate that they have previously encountered those words during the LTM learning phase. To control for saliency effect of the red color, we presented two successive new words in the *new* condition in red as well. These red-highlighted words could appear at any two possible positions within the list in a counterbalanced fashion, mimicking the distribution pattern of the *word pairs* and *singletons* conditions.

This time, all trials were intermixed across the experiment, rather than being organized in blocks by condition.

### Data analysis

The data were analysed equivalently to Experiment 1, but this time the fixed-effect of serial position included an additional level of serial positions 6 and 7 [*1,2*; *2,3*; *3,4*; *4,5*; *5,6; 6,7*] and the fixed-effect of word type included the highlighted red words and neutral black words [*red, black*]

### Results

**Figure 4:**
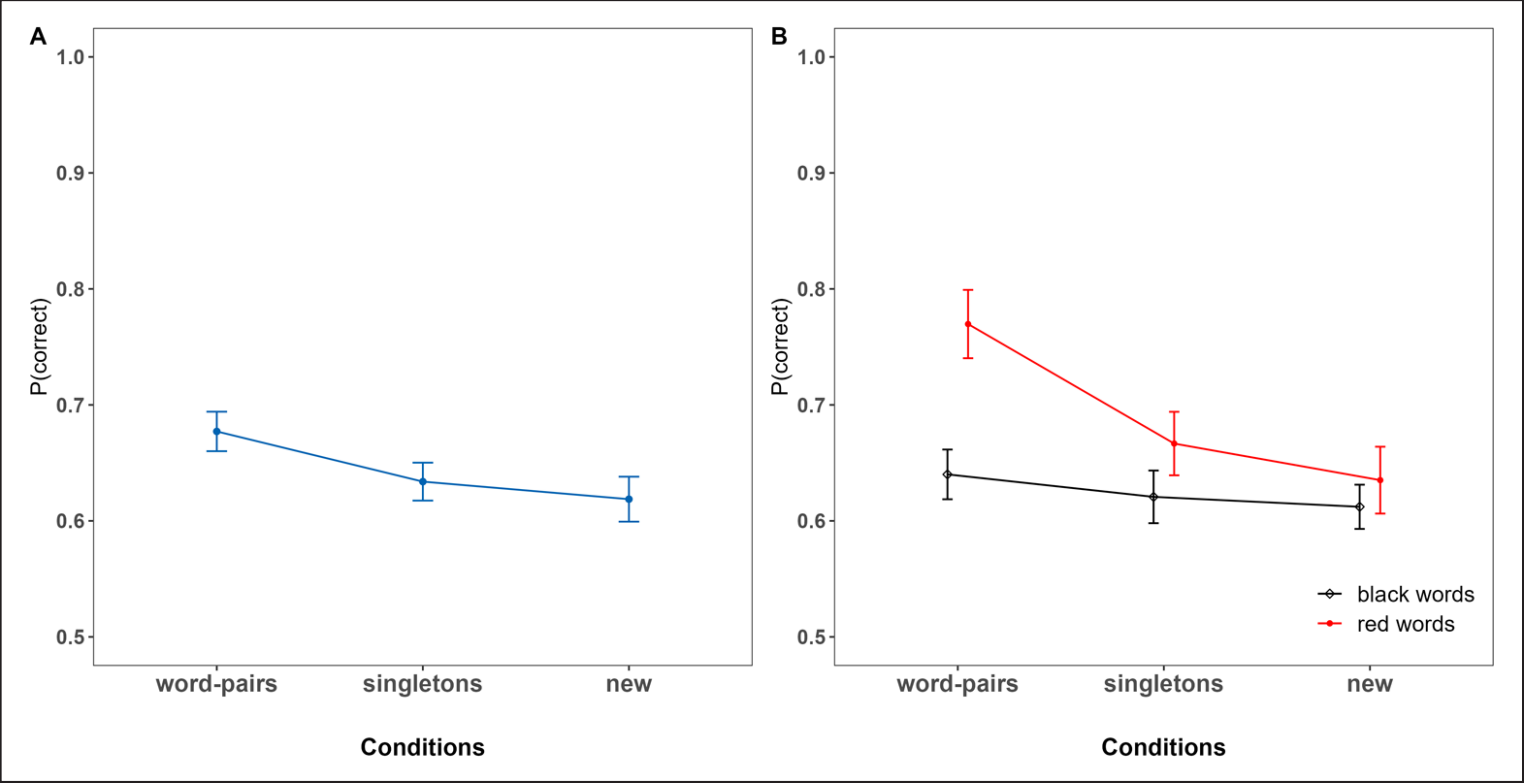
Proportion of immediate Recall Performance as a Function of Condition. **Panel A:** overall Performance. **Panel B:** Performance for highlighted red Words and new Words presented in black. *Note*. In both the *word pairs* and *singletons* conditions, the red words represent the LTM words. In the *new* condition, however, the red words indicate new words highlighted in color red to establish a baseline and control for any potential salience effects.

Figure 4 shows overall performance across (A) conditions and (B) distinguishing performance depending on the color in which the words were introduced.^3^ In contrast to Experiment 1, this time the advantage of LTM information over new information was found not only within the *word pairs* condition (BF_10_ = 1.82 × 10^5^ [6.08 × 10^4^ – 4.79 × 10^6^]), but also within the *singletons c*ondition (BF_10_ = 3.21 [1.60 – 5.72]) (see Figure 4B). The latter reflected an item activation boost for LTM words (yet which were not associated within the same episode). Importantly, there was no effect of saliency when comparing red *versus* black words within the *new* condition (BF_10_ = 0.21 [0.11 – 0.50]).

**Figure 5:**
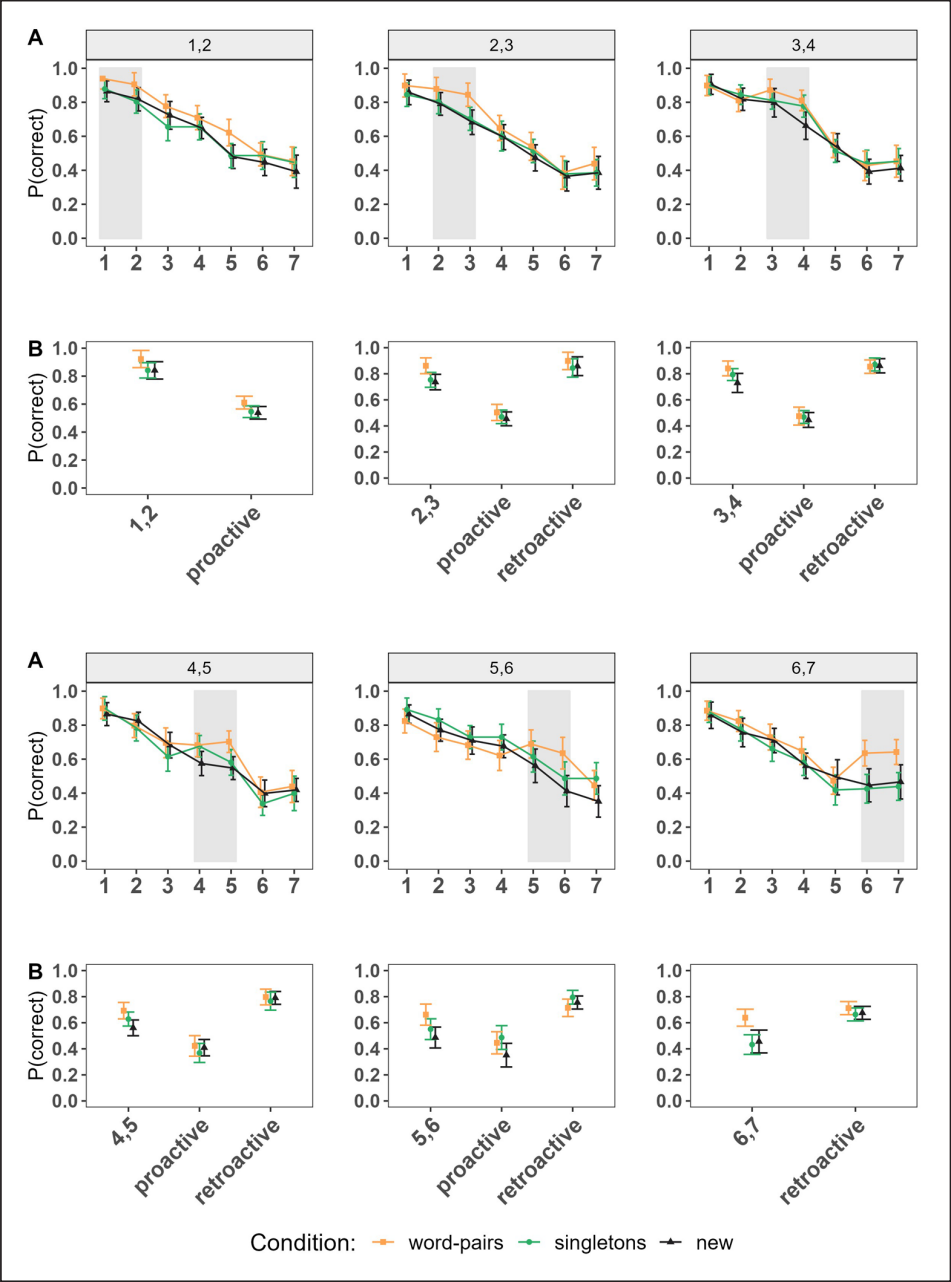
Experiment 2. **Panel A:** Serial Position Curves depending on the Serial Positions in which the highlighted Words were introduced within the list. **Panel B:** Performance of highlighted Words at the Serial Positions of interest; proactive and retroactive performance. *Note*. The numbers (e.g., 1,2) denote the serial positions at which the highlighted red words were introduced within the list. They correspond to LTM words in both LTM conditions, and to the matching positions of words in the *new* condition. Proactive and retroactive effects indicate the aggregated performance of words presented in serial positions following or preceding the introduction of highlighted words, respectively.

Based on the serial position curves from Figure 5A, we now compare performance *between* conditions of LTM versus new information, and to test proactive and retroactive effects on new information based on the position of LTM words within the list (see Figure 5B). First, and supported by the statistical results presented in Table 2, immediate memory for words from pre-learnt word pairs were remembered consistently and credibly better at all serial positions compared to words of the *new* condition presented at the same serial positions. Moreover, compared to the *singletons* condition, immediate memory for LTM words from the *word pairs* conditions was credibly superior at the beginning and at the very end of the list (i.e., positions 1 and 2, 2 and 3, and 6 and 7). In contrast, there were no credible differences between *singletons* and words of the *new* condition at any serial positions within the list.

**Table 2:** Experiment 2. Bayes factors (BF_10_) of the pairwise comparisons between conditions for each serial position in which the highlighted red words were introduced in the lists.


	*WORD PAIRS VS SINGLETONS*	*WORDS PAIRS VS NEW*	*SINGLETONS VS NEW*

*Serial positions 1,2*	**22** [12.40–78.13]	**116.80** [58.17–704.42]	.14 [0.08–0.32]

*Proactive*	0.96 [0.53–1.79]	1.36 [0.78–3.19]	0.08 [0.04–0.19]

*Retroactive*	–	–	–

*Serial positions 2,3*	**3.87** [1.52–20.47]	**72.09** [21.50–572.80]	0.17 [0.04–0.15]

*Proactive*	0.08 [0.04–0.18]	0.11 [0.06–0.26]	0.06 [0.04–0.15]

*Retroactive*	0.24 [0.17–0.38]	0.21 [0.14–0.34]	0.18 [0.11–0.35]

*Serial positions 3,4*	0.32 [0.14–1.97]	**13.47** [5.03–71.59]	0.58 [0.36–0.92]

*Proactive*	0.06 [0.03–0.13]	0.08 [0.04–0.14]	0.08 [0.04–0.18]

*Retroactive*	0.09 [0.06–0.19]	0.08 [0.05–0.18]	0.12 [0.07–0.24]

*Serial positions 4,5*	0.32 [0.14–1.82]	**11.89** [5.38–96.11]	0.37 [0.21–0.72]

*Proactive*	0.14 [0.08–0.34]	0.06 [0.04–0.14]	0.14 [0.09–0.30]

*Retroactive*	0.10 [0.06–0.19]	0.07 [0.04–0.17]	0.10 [0.06–0.22]

*Serial positions 5,6*	1.67 [0.77–9.39]	**37.39** [13.18–294.45]	0.18 [0.11–0.38]

*Proactive*	0.10 [0.06–0.21]	0.45 [0.27–1.01]	2.22 [1.64–2.50]

*Retroactive*	0.29 [0.20–0.45]	0.09 [0.05–0.20]	0.12 [0.07–0.25]

*Serial positions 6,7*	**42.34** [18.05–224.15]	**23.64** [9.61–117.43]	0.10 [0.06–0.21]

*Proactive*	–	–	–

*Retroactive*	0.23 [0.14–0.52]	0.09 [0.06–0.23]	0.08 [0.05–0.21]


*Note*. Serial positions represent the positions in which the LTM words were introduced in the *word pairs* and *singletons* conditions, which are compared to the words presented at the same serial positions of the *new* condition. Credible BF’s are printed in bold.

Regarding proactive and retroactive effects, our findings indicate once again that the observed improvement in performance for LTM words did not translate into an advantage for the performance of novel words presented either prior or subsequently to the LTM words compared to the *new* condition, irrespective of the position in which the latter were introduced within the list.

### Discussion

The main goal of Experiment 2 was to investigate the possibility that participants were unable to recognize the matching episode at encoding due to the lack of a 1-to-1 correspondence in the structure of the WM representations – subsequently preventing any effects of freeing WM capacity. To achieve this, we decided to guide and facilitate awareness of LTM information, by highlighting LTM words in red and informed participants that this could be indicative of words they had pre-learnt during the LTM learning phase.

First, our results showed that LTM words of the *word pairs* condition had a better overall performance compared to the *singletons* and *new* condition, which replicates the observed pattern in Experiment 1.

Second, manipulating the saliency of the information by making participants aware that the color was informative, not only resulted in consistently superior performance of LTM words compared to new words *within* the *word pairs* condition, but also extended to LTM singletons. However, when comparing performance of LTM words to words of the *new* condition at all the same serial positions, our results revealed superior performance of LTM words in the *word pairs* condition regardless of their serial positions within the list. This was not observed when comparing LTM singletons to words of the *new* condition at any serial position.

While the saliency manipulation boosted performance through item activation of the singleton LTM words compared to new words in the same list (local effect), immediate performance for sequentially presented singletons did not exhibit an advantage when compared to new words of the *new* condition at any serial position (global effect). This indicates once again that superiority of performance for LTM words, both locally and globally, is more robust only when both LTM words form part of a pre-learnt word pair, consistent with Experiment 1.

Third, although memory for the LTM words themselves was improved, our results again provided no evidence of freeing WM capacity. Instead, they are consistent with a redintegration account, when assuming that this process is more likely to occur for intact episodes rather than single words. The absence of any advantage for new information in lists containing LTM words suggests that participants did not reduce WM load when LTM information was included, not even in the form of pre-learnt word pairs. This lack of freeing-up capacity indicates that information was probably not successfully recoded or offloaded during encoding, not even when the information was presented at the very beginning of the list. This stands once again in contrast to previous research using semantic LTM representations (Portrat et al., 2016; Thalmann et al., 2019) or larger pre-learnt lists of at least three elements (Norris et al., 2020).

We argue that the creation and maintenance of positional bindings in intermixed lists for both, LTM and new words, is crucial for successfully completing the serial recall task. This is particularly relevant in the context of LTM representations lacking positional information, especially for paired associates with symmetrical properties (Kahana & Caplan, 2002), where no ordinal or positional information is inherent to the representations. As a consequence, the implementation of any chunking strategy during encoding or purely relying on the LTM representations (i.e. offloading) is not sufficient to successfully solve the serial recall task.

Beyond the demand of encoding positional bindings, an additional challenge may arise when pre-learnt word pairs must be encoded separately without structural support at WM encoding. Such support can be observed when the word pairs are presented simultaneously (Bartsch & Shepherdson, 2022, 2023; Chen & Cowan, 2005, 2009), or when a grouping structure is used. Previous studies have achieved this by organizing the information spatially, for example, by placing the LTM information in separate rows from new lists (Musfeld et al., 2024; Thalmann et al., 2019).

Therefore, the utilization of a clearly defined structure that can be employed during both encoding and retrieval may facilitate not only the recognition of the LTM representation, but also might serve as a template for recalling information that already exists, allowing for a more efficient reallocation of resources and freeing of capacity in WM. To test this hypothesis, we ran Experiment 3.

## Experiment 3

In this experiment, we aimed to test whether the structural congruence between a visuo-spatial template at encoding and at recall would facilitate not only the recognition of episodic LTM representations but would also allow participants to more efficiently engage in chunking or offloading strategies, thereby freeing-up resources for processing novel information. Specifically, our goal was to determine if the support of a visuo-spatial structure in WM would contribute to a more efficient organization and encoding of the information. We aimed to test if the visuo-spatial support contributed to a reduction in WM load when LTM words were included, even when the core of the task requires the creation of positional bindings for each item.

To address this, in Experiment 3, we focused only on matching episodic representations (*word pairs* condition) and dropped the *singletons* conditions as only the former led to consistent benefits on WM performance in our previous two experiments.

**Figure 6:**
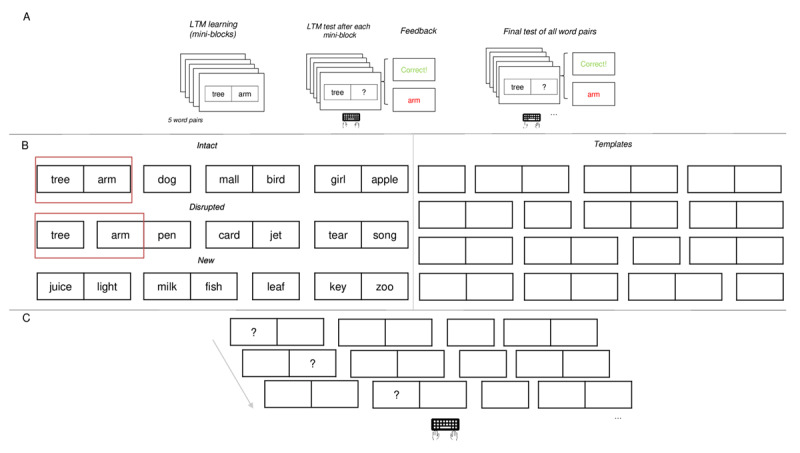
Events representing the Procedure of Experiment 3. **Panel A:** LTM learning Phase. **Panel B:** on the Left Side, WM encoding phase; three different Conditions from Top to Bottom: **(a)** LTM Intact Structure Condition **(b)** LTM Disrupted Structure Condition **(c)** New Condition; on the Right Side, the different Templates used to create the Conditions; Panel C: WM testing Phase: Typed Serial Recall.

For this experiment, the word pairs in the learning phase were presented in two adjacent boxes, forming a cohesive visual group. For the serial recall task, we presented a visuo-spatial template consisting of seven boxes arranged horizontally and centered on the screen. LTM words were presented sequentially in either intact pairs (boxes were adjacent) or disrupted pairs (boxes were spatially separated by a gap, see Figure 6B).

By manipulating the spatial arrangement of the WM template to match or disrupt the structure of the LTM word pair associations, we aimed to test the impact of structural grouping congruence on the efficiency of serial recall.

### Method

#### Participants

An independent sample of 36 participants (M_age_ = 29.9 years), who indicated English as their first language, being from English speaking countries and successfully learnt at least 70% of the word pairs presented during the initial phase of the experiment were recruited online via Prolific (approval rate between 90–100). The sample size was determined by the number of necessary conditions to counterbalance the design, as described below.

#### Materials and procedure

The general procedure of Experiment 3 was the same as for the previous experiments with the following changes: First, for the long-term memory phase, participants were required to learn 20 word-pairs. This time each word pair was presented in two adjacent boxes centered on the screen (see Figure 6A).

Second, for the WM task (Figure 6B), instead of presenting words one by one at the center of the screen, a visuo-spatial template with seven horizontally arranged boxes was displayed in the middle of the screen. Words were presented sequentially from left to right within the boxes, each for 1000 ms, followed by a 500 ms ISI. Critically, we designed four different visuo-spatial templates to create conditions in which the LTM words^4^ were either presented in the same encoding structure they were learnt, (*intact* condition – two adjacent boxes-); or in a disrupted structure, with the words presented in two non-adjacent boxes separated by a gap (*disrupted* condition). Given the complexity of the design, we focused on specific serial positions within the list: the onset (positions 1 and 2), the middle (positions 3 and 4, and 4 and 5), and the end (positions 6 and 7) of the list. As depicted in the right panel of Figure 6B, for the disrupted structure, only one of the four templates could be linked to one of the four serial positions of interest (e.g., only the first template could be associated to disrupted serial positions 1 and 2). However, in the intact structure, there were instances where more than one visuo-spatial template could be associated with one of the serial positions (e.g., three out of four templates for serial positions 1 and 2).

To maintain a balanced design, each participant was assigned a single template for the *intact* presentation of LTM words at each specific serial positions. For example, participant X would always encounter the fourth template at positions 1 and 2, the third template at positions 3 and 4, the second template at positions 4 and 5, and the first template at positions 6 and 7.

A total of thirty-six participants were required to account for all the possible template-serial position combinations.

Each participant completed 10 blocks, with each block comprising 12 trials in random order, which consisted of four for each LTM condition (*intact* and *disrupted*), corresponding to the four serial positions of interest. An additional four trials were included for each template, featuring only new words (*new* condition), serving as our baseline.

After presenting all the words on the screen, participants completed an immediate serial recall test. The same template used at encoding appeared on the screen at test. A question mark used as a cue appeared at the first box, prompting participants to recall the first word from the list – the word that was presented within that box. After entering their response, participants pressed the “Enter” key to proceed to the next box and a new interrogation mark cued the following box. This process continued until the final box (see Figure 6C).

#### Data Analysis

The data were analysed equivalently to Experiment 1 and 2, but with a focus on differences in leveraging LTM between episodic representations presented in visuo-spatial templates that were congruent or incongruent with the learning phase (*intact* and *disrupted* conditions, respectively).

Hence, to test whether there were credible differences between the LTM words and new words *within* both LTM conditions (*intact* and *disrupted*), we fit a model were condition [*intact, disrupted and new*] and word type [*LTM, new*] were included as fixed-effects, and then computed the conditional effects for the pairwise comparisons of interest (LTM vs. new words in both the *intact* and *disrupted* conditions). This allowed us to test whether an immediate performance benefit for LTM words over new words within the same trial was due to a *local* advantage for episodic representations presented in an encoding structure that matched the one of the learning phase (benefits only for LTM words in the *intact* condition) or whether there was a benefit of lists including LTM information irrespective of the encoding structure in which they were introduced in WM (benefits for both LTM words in the *intact* and *disrupted* conditions).

To test whether there were differences *between* conditions at each serial position of interest, as proactive and/or retroactive effects, we fit three independent models using serial positions [*1,2*; *3,4*; *4,5; 6,7*] and condition [*intact, disrupted and new*] as fixed-effects. Then we computed the conditional effects for the pairwise comparisons of interest that will be described next.

The first model aimed to compare performance of LTM words of the *intact* and *disrupted* conditions to words of the *new* condition at the same serial positions in which the LTM words were introduced. This allowed us to test whether an immediate performance benefit for LTM words over new words between conditions was due to a *global* advantage for episodic representations presented in a congruent encoding structure to that of the learning phase (an advantage for LTM words in the *intact*) or to LTM words irrespective of the encoding structure, in which they were presented in WM (an advantage for both LTM words in the *intact* and *disrupted* conditions over words from the *new* condition at the same serial positions).

The second and third models aimed to test whether including LTM words in trials of the serial recall task reduced WM load, depending on the encoding structure in which they were presented and where the LTM words were introduced within the list. Specifically, the second model tested whether there was a proactive benefit for words presented *following* the presentation of LTM words and the third model aimed to test whether there was a retroactive benefit for words introduced *preceding* the presentation of LTM words.

### Results

**Figure 7:**
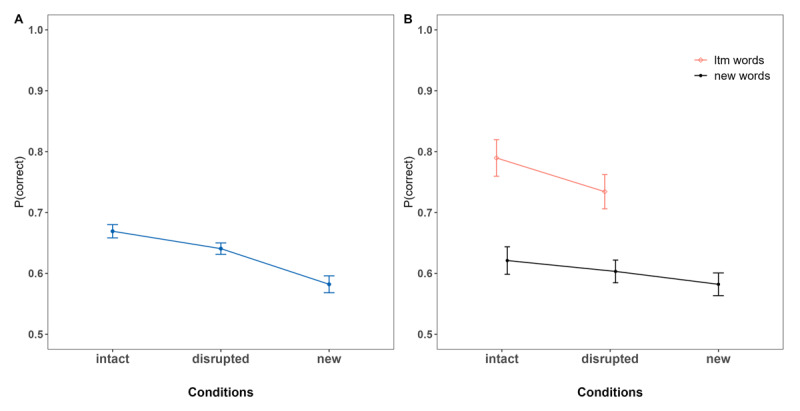
Proportion of immediate Recall Performance as a Function of Condition. **Panel A:** overall Performance. **Panel B:** Performance for LTM Word Pairs and new Words for LTM Conditions.

Figure 7 depicts the proportion of correct responses as a function of (A) condition and (B) performance for LTM and new words independently within both LTM conditions (*intact* vs. *disrupted*). As shown in Figure 7B and supported by the analysis, performance of LTM words was credibly higher compared to new words *within* trials irrespective of the encoding structure in which the LTM words were introduced in the WM templates (*intact* condition: BF_10_ = 2.06 × 10^6^ [1.72 × 10^5^ – 1.47 × 10^7^] ; *disrupted* condition: BF_10_ = 1.43 × 10^5^ [3.80 × 10^4^ – 7.36 × 10^5^]).

**Figure 8:**
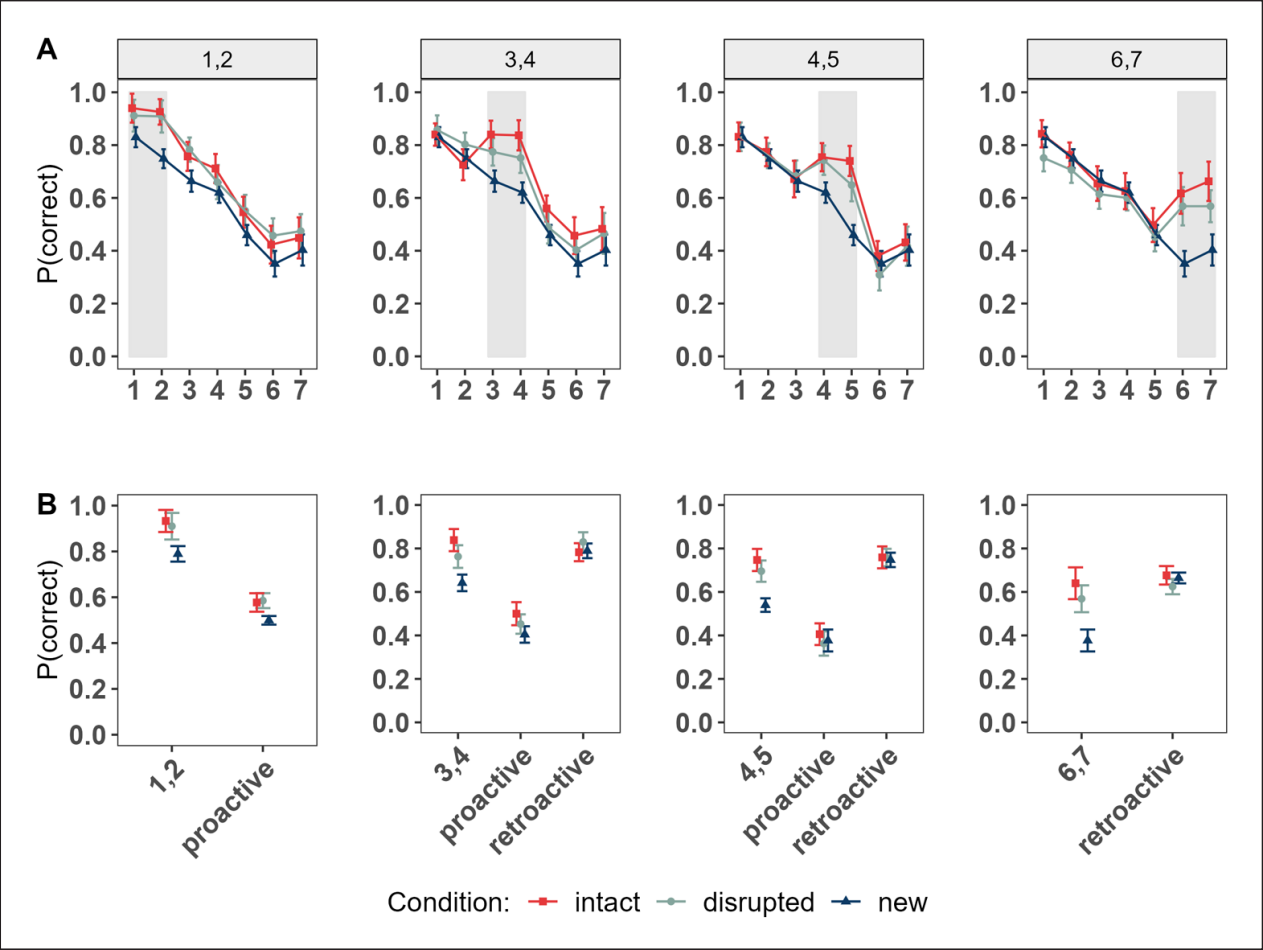
Experiment 3. **Panel A:** Serial Position Curves depending on the Serial Positions in which the LTM word pairs were introduced within the List. **Panel B:** Performance of Words at the Serial Positions of interest (LTM word pairs in the intact and disrupted Conditions; Words at matching Positions of the new Condition); proactive and retroactive Performance. *Note*. The numbers (e.g., 1,2) denote the serial positions at which the LTM word pairs were introduced within the list in both LTM conditions, and correspond to the same positions of words in the *new* condition. Proactive and retroactive effects indicate the aggregated performance of words presented in serial positions following or preceding the introduction of LTM word pairs, respectively. These specific serial positions are the same for words in the *new* condition.

We next focused on performance between conditions for LTM word pairs and words from the *new* condition based on the position of word pairs within the lists (see Figure 8). As shown in Figure 8B and supported by the statistical results presented in Table 3, we consistently found superior immediate memory performance for LTM word pairs compared to words in the *new* condition at all serial positions (beginning, middle and end of list), and regardless of the encoding structure (*intact* or *disrupted*).

**Table 3:** Bayes factors (BF_10_) of the pairwise comparisons of new words between conditions in Experiment 3.


	*INTACT VS DISRUPTED*	*INTACT VS NEW*	*DISRUPTED VS NEW*

*Serial positions 1,2*	0.15 [0.08–0.45]	**1.67 × 10**^5^ [1.96 × 10^4^ –2.02 × 10^6^]	**5008.62** [2.55 × 10^3^ –1.88 × 10^4^]

*Proactive*	0.04 [0.02–0.10]	**15.71** [8.83–47.44]	**401.45** [1.69 × 10^2^–2.34 × 10^4^]

*Retroactive*	–	–	–

*Serial positions 3,4*	**5.51** [3.65–14.40]	**5.41** × **10**^5^ [3.54 × 10^4^–2.54 × 10^7^]	**156.06** [57.31–4094.10]

*Proactive*	0.26 [0.15–0.52]	**28.13** [16.66–51.10]	0.52 [0.32–1.06]

*Retroactive*			

*Serial positions 4,5*	0.44 [0.27–1.87]	**1.57** × **10**^5^ [1.26 × 10^4^–1.72 × 10^6^]	**772.92** [432.20–5198.13]

*Proactive*	0.16 [0.09–0.33]	0.08 [0.05–0.20]	0.05 [0.03–0.13]

*Retroactive*	0.05 [0.03–0.12]	0.06 [0.04–0.15]	0.05 [0.03–0.12]

*Serial positions 6,7*	0.67 [0.42–2.51]	**2.56** × **10**^5^ [4.50 × 10^4^–9.12 × 10^6^]	**6217.68** [1060.70–1. 60 × 10^6^]

*Proactive*	–	–	–

*Retroactive*	1.50 [0.90–2.85]	0.05 [0.03–0.12]	0.89 [0.56–1.61]


*Note*. Serial positions represent the positions in which the LTM words were introduced in the *intact* and *disrupted* conditions, which are compared to the words presented at the same serial positions of the *new* condition. Credible BF’s are printed in bold.

While descriptively it appears that the intact encoding structure enhanced performance of word pairs compared to those encoded in a disrupted structure, the only credible difference in which memory performance for word pairs of the *intact* condition was higher compared to those of the *disrupted* condition was when they were presented at serial positions 3 and 4. This suggests that the benefits of presenting a 1-to-1 matching structural representation to that in which the episode was learnt are most pronounced at the beginning of the list. However, presenting information at the very beginning (i.e. the edge) of the list leads to equivalent performance independent of the visuo-spatial arrangement in which the LTM pairs were introduced.

Notably, for the first time in these set of experiments, we observe a benefit for new words when LTM pairs were introduced within a list – indicating freeing of capacity. When the LTM information was presented at the very beginning of the list, we observed a proactive memory benefit for new words (compared to the new words of the *new* condition at the same serial positions), irrespective of the encoding structure (*intact vs. disrupted;* see Table 3*)*. For the *intact* condition, this proactive benefit was also present when the LTM pair was presented at positions 3 and 4, but no longer for the *disrupted* condition. When the LTM pair was presented towards the end of the list, no proactive effect was present regardless of the format. None of the conditions produced a retroactive benefit (see Table 3).

### Discussion

Our main objective in Experiment 3 was to test whether incorporating LTM word pairs in a serial recall task, using a visuo-spatial configuration in WM that matched the learning phase, would not just ease recognizability and recall of the LTM word pairs, but also reduce WM load.

To achieve this, we used visuo-spatial templates wherein the LTM word pairs could be presented adjacent to each other forming a group (*intact* condition) – while still being sequentially presented. This mirrored the structure used during the learning phase. We contrasted this with a disrupted structure, where the two words forming the word pair were visuo-spatially separated.

Our results revealed that performance of LTM word pairs was consistently higher compared to new words *within* the same lists, regardless of whether the encoding structure in the template matched the pre-learnt LTM representations (*intact* condition) or whether it was disrupted. Moreover, when compared to words of the *new* condition, LTM word pairs showed superior performance across all the matching serial positions (beginning, middle, and end of the list).

Furthermore, the utilization of visuo-spatial templates during both encoding and recall in WM not only resulted in benefits for the LTM words themselves but also contributed to reducing the load on WM, particularly when LTM pairs were introduced at the very beginning of the list in both *intact* and *disrupted* encoding structures. This advantage only extended further to conditions in which the LTM pair was presented later in the list when the encoding structure was *intact*, but not when it was *disrupted*.

Compared to Experiment 1 and 2, this experiment created a context in which information was visually organized at encoding. This manipulation not only facilitated the recognition of LTM information and enhanced its immediate recall, but also contributed to freeing-up WM capacity for subsequent new words, although only when WM was not yet loaded. This is consistent with previous findings using semantic LTM representations (Mızrak & Oberauer, 2021; Portrat et al., 2016; Thalmann et al., 2019).

The fact that the proactive effects were observed for both *intact* and *disrupted* conditions – in case the word pairs were presented at the beginning of the list – suggests that an identical matching encoding structure may not play a major role at this edge position within the list. This is because the information presented at the beginning of the list is inherently distinct and salient (Burgess & Hitch, 2005; Henson, 1999). However, the observed advantage of the *intact* structure over the *disrupted* one, at later positions (3 and 4) speaks to the benefits of encountering a matching visually structured episodic representation. This format most likely facilitated recognition of prior learnt representations and enabled a more effective engagement in chunking or offloading strategies to release resources for upcoming information, as evidenced in our results.

The revealed proactive benefits shown here, are in line with the assumption that LTM information would aid in the efficient processing of new stimuli once it has been chunked in LTM, by which redundant information is removed from WM and does not interfere with upcoming information (Mızrak & Oberauer, 2022; Norris et al., 2020; Norris & Kalm, 2021; Thalmann et al., 2019). This could entail a process such as offloading the second word associated with the first one of the word pair, probably after both elements have been encoded. We further argue that fully offloading the word pair is unlikely, because of the reason mentioned above: the task requires the creation of positional bindings to solve the task, as at least one element needs to be bound to the specific context (the position within the list).

As suggested by Thalmann, et al. (2019), when the LTM pair appears later within the list, previously encoded representations may be adversely affected by the encoding of subsequent LTM elements before they can undergo the chunking process. Consequently, as the list lengthens and more information is retained in WM, the potential harm increases, thereby impeding the ability of LTM information to mitigate this damage.

These results indicate overall that the way we structure and encode the information in WM is an important factor that regulates the effectiveness of LTM contributions to WM.

## General Discussion

The present study investigated the interaction between WM and pre-learnt episodic LTM item-item representations in a serial recall task. It focused on how WM can flexibly utilize episodic LTM information, and on the role of recognizability and structuring the episodic LTM information at WM encoding for the benefit to occur.

Across three experiments, we studied the extent to which incorporating LTM word pairs at different positions within the lists, which encompass item-item representations, would benefit WM serial recall, which requires the creation of item-position bindings (Burgess & Hitch, 1999; Henson, 1999). We expected this benefit to occur in two ways: (1) through better immediate memory for LTM pairs compared to new information – within lists and compared to the same serial positions of words consisting of just novel lists, and (2) by freeing-up WM capacity.

To properly analyze the effect of freeing-up WM capacity, we employed an unbiased approach by specifically comparing the serial positions that preceded or followed the introduction of LTM words to the exact same serial positions in the baseline condition (*new* condition), rather than comparing them to the aggregated performance across all serial positions in the baseline. This method allowed us to avoid the risk of masking potential effects that might be overlooked if all serial positions were included in the comparison. Specifically, we control for particularities of serial position curves, such as primacy or recency effects.

In Experiment 1 we found that participants benefited from the episodic prior knowledge that retained the contextual binding information (word pairs) and not from item activation when two words from different episodes were included. This advantage was reflected as better immediate recall for LTM word pairs over new information within trials and compared to new words from lists consisting of entirely new words at almost all of the serial positions. However, the advantage of including LTM information in mixed lists did not extend to improving recall of new words within the same lists, indicating that WM capacity was not freed-up during encoding when LTM information was included. This is inconsistent with the idea that participants utilized offloading or chunking strategies for pre-learnt word pairs, as we did not observe any evidence for freeing up of WM capacity. Instead, the benefits of including LTM information in serial recall are more consistent with a redintegration account. In this process, the information is encoded as originally presented in WM and pre-existing LTM information helps to reconstruct the WM traces during testing. However, our findings suggest that such a redintegration process does not purely rely on the activation of specific items in LTM, but also on the availability of intact episodic information.

Results from Experiment 2 revealed a similar pattern, even when we enhanced awareness of the LTM information during encoding by manipulating the saliency of LTM words. Overall, facilitating recognition of the LTM information improved recall of LTM words (both word pairs and singletons) compared to new words *within* trials (locally). However, this advantage was not as robust for singletons, as their benefit did not extend to comparisons with the baseline (globally). This finding reiterated that performance of remembering LTM words was consistently superior when both words matched an already stored episodic representation, rather than arising from an item activation account. Once again, no freeing-up of WM capacity was observed and the observed LTM benefits in immediate recall are best explained by WM traces being redintegrated at recall from pre-learnt episodes.

A reduction in WM load was however observed in Experiment 3, when we introduced a visuo-spatial configuration in the WM task, facilitating recognition of LTM information and in turn, facilitating the utilization of chunking strategies. Using intact encoding structures that mirrored the ones present in the learning phase (by presenting the words in adjacent boxes) extended the proactive benefits to later serial positions of where the LTM words could be presented compared to templates where that structure was disrupted by visuo-spatially separating the sequential presentation of the pair.

The freeing-up of WM capacity observed in Experiment 3 could be attributed to the exclusion of the *singletons* condition (in which two words came from different pre-learnt pairs). By presenting only conditions where memoranda matched pre-learnt episodes rather than mismatching, the information may have been more predictable and reliable, enabling more effective encoding strategies—such as encoding only the first word of each pair instead of waiting for the second to confirm a match. However, this explanation is challenged by Experiment 1, where conditions were presented in blocks. In that setup, the *word pair* condition was also predictable within a block, yet no freeing-up of WM capacity was observed. This suggests that the benefit arised not merely from predictability but from having a visuo-spatial encoding structure that both supports LTM representations and reduces WM load, facilitating the processing of novel information.

### Benefits of episodic LTM to WM

The consistent finding across these and previous experiments incorporating prior knowledge in the form of episodic LTM representations in WM tasks, is the emergence of a clear benefit for LTM representations over novel information within the same lists (Bartsch & Shepherdson, 2022, 2023; Norris et al., 2020; Thalmann et al., 2019). In our case, this mainly arose in case the sequentially presented LTM words consisted of a pre-learnt episodic association in the context of serial recall.

However, despite the consistent advantage for recalling pre-learnt information over newly presented information, we did not consistently observe freeing of WM capacity for new words. Why is a capacity-freeing effect not consistently observed across different WM tasks?

Considering our foundational understanding of WM as a limited capacity system primarily focused on holding relevant information to fulfill current demands (Cowan, 2008; Oberauer, 2009), the goal of the WM task itself becomes crucial in regulating the system’s flexibility in relying on prior information. Granting access to LTM information through a flexible gate, as previously suggested (Bartsch, Frischkorn, et al., 2024; Bartsch & Shepherdson, 2022; Mızrak & Oberauer, 2022; Oberauer et al., 2017), therefore must hold benefits for solving the task at hand – over and above the costs that might arise from sources of interference. Therefore, when previously learnt information contains the necessary bindings to accomplish the WM task at hand and can be reliably retrieved during recall, drawing on LTM should be advantageous. However, when such bindings are not inherently included in the LTM representations, WM must create the necessary temporal bindings of information to the context from scratch (Oberauer, 2019).

In the present study, the pre-learnt item-item associations, represented as word pairs, would have required additional bindings to the context to successfully complete the serial recall task. Therefore, the LTM words may not have undergone a chunking or offloading process, preventing freeing of WM capacity. Still, even in this situation, a benefit for the LTM information itself could nevertheless arise. For instance, the existing LTM representations could help to reconstruct the WM trace at recall, in case the trace was degraded (Botvinick, 2005; Hulme et al., 1997; Norris et al., 2020; Thorn et al., 2005) compared to weaker representations of newly encountered information that have no support of pre-learnt episodes.

Indeed, when inspecting previous tasks in which a capacity-freeing effect of LTM was observed, we find that creating bindings from scratch for information included in WM was not inherently necessary. This is the case for tasks which did not require the formation of new associations to the context, such as recalling the paired word in a cued-AFC task (Bartsch & Shepherdson, 2022, 2023); or in cases in which the task required the creation of *fewer* bindings (Chen & Cowan, 2005, 2009). In the latter studies, when pre-learnt word pairs were presented simultaneously in pure LTM pair lists, recognizing the pair might have allowed to only encode and bind the first word to its positional context. For the second word, no additional binding was required, as it could be recalled based on its association to the first word during recall (Chen & Cowan, 2005, 2009). Lastly, other previous tasks used an encoding format which allowed for chunking strategies at recall, such as presenting chunks as independent lists on separate rows of the screen (Thalmann et al., 2019). Here, retrieval of the full list only required encoding of the first word (always associated with serial position 1) of the LTM list. Hence, the need to create new bindings for all the elements within WM may have been circumvented.

### Encoding structure matters

In the present study, a reduction in memory load emerged when the serial recall task was supported by the utilization of visuo-spatial templates to organize the to-be-encoded information. Recent findings have highlighted the importance of encoding structures for learning repeated lists: they facilitate the encoding of information into an integrated representation (Musfeld et al., 2024) and changes in the structure at encoding have been shown to have detrimental effects in learning new associations (Bower & Winzenz, 1969; Musfeld et al., 2024).

We argue that the use of such structures facilitates not only awareness of matching pre-learnt episodes – a necessary, though not sufficient step, as demonstrated in Experiment 2 – but also a more efficient way to rely on visuo-spatial templates for better reallocation of resources, therefore a reduction in WM load when encountering episodic LTM representations. This, importantly, depended on *where* the information was presented within the list, and was more pronounced when the visuo-spatial template matched the representation in which the LTM word pairs were acquired (*intact* condition). This pattern aligns with Thalmann et al.’s (2019) findings, where proactive rather than retroactive effects are observed using semantic chunks: the benefits of memory load reduction appear when WM capacity has not been exceeded. This is because encoding the elements of the word pair individually after new words have already been encoded could damage these representations, as earlier presented items may be more degraded and susceptible to interference. However, engaging in a chunking strategy earlier in the list for LTM information allows for a reduction in WM load, as long as the information can be chunked or offloaded – for serial recall, presumably partially offloaded. Having the visuo-spatial template as additional support allowed participants to not only rely on item-position bindings but also to derive extra benefits from the spatial context. In this case, the easier an episode is recognized, the easier participants can chunk the elements, and the less resources are required. Hence, more resources will be freed-up and are available to items following from pre-learnt LTM pairs. This is consistent with the suggestion by Norris et al. (2020) that the efficiency of engaging in chunking strategies is dependent on the costs of recoding or offloading LTM information during encoding. However, our findings show that these costs are not only dependent on the size of the chunk as shown by Norris et al. (2020), but also on the ease of recognizing and matching an existing LTM representation to the current encoding context.

## Conclusion

The present study highlights the critical role of how information is structured at encoding for benefits of episodic LTM to WM to flexibly arise. While including LTM words in serial recall can facilitate the immediate memory for words matching pre-learnt pairs themselves, benefits in recalling new stimuli within the same lists depends on whether the LTM information can be chunked efficiently given the support of the encoding structure. Finally, our analysis underscores the importance of comparing corresponding serial positions between LTM lists and new lists for detecting effects of interest.

## Data Accessibility Statement

The data and the analysis scripts can be accessed on the Open Science Framework (https://osf.io/r8tyc/).

## Footnotes

So far, offloading has been found in tasks where clear cues are provided during test to retrieve the LTM information, such as in alternative-forced choice tasks. However, it remains unclear whether this mechanism also operates in serial recall. One possibility is that the serial position in which the LTM information was introduced serves as the cue to retrieve the pre-learnt LTM information. During recall, the information with the highest familiarity signal from LTM given the cue could then be reinstated at those serial positions, even in the absence of explicit encoding in WM. Therefore, offloading in serial recall would depend on the position as a cue to guide retrieval from LTM.

Here we interpret the benefits of LTM word pairs as a result of retrieving the full pre-learnt episodes. However, an alternative explanation is that better recall of pre-learnt word pairs in WM trials could also occur from learning processes in the linguistic system, such as changes in the co-occurrence probability of specific items. While these factors could play a role in memory performance beyond the retrieval of prior episodic information, disentangling their specific contributions to the benefits of pre-learned word pairs is beyond the scope of this study.

Note that in Figure 4B we distinguished the word type by color. Specifically, the red words represent the LTM words, and the black words the new information in both the *word pairs* and *singletons* conditions. However, the red words in the *new* condition represent exclusively words highlighted in red to control for the saliency effect of the color.

LTM words refer just to word pairs for this experiment.
